# The Philosophic Relation of Homœopathy to Surgery

**Published:** 1892-11

**Authors:** T. D. Stow

**Affiliations:** Mexico, N. Y.


### THE PHILOSOPHIC RELATION OF HOMŒOPATHY TO SURGERY.

T. D. Stow, M. D., Mexico, N. Y.

Fellow Associates:—Our mutual friend and co-worker, the Chairman of the Bureau of Homœopathics for 1891–92, Dr. B. Fincke, persuaded me to report a paper of the foregoing title at this the 1892 session of the I. H. A. I am not a little sorry to have undertaken the job, for, before this article was finished, I wished the job had been let out to some one more capable of doing the subject justice. A paper of this kind involves more than at first sight seems evident, for there are divergent views entertained in this Association as to the scope of papers relating to the Bureau of Surgery, as well as to the character of articles addressed to the Bureau of Homœopathics. This organization has been effected for a specific purpose, that purpose being clearly stated in the declaration of principles.

*First.*—The law of similars is the law of cure.

*Second.*—A proper knowledge of the curative powers of medicines is derived from provings made upon healthy persons.

*Third.*—Hahnemann's *Organon of the Healing Art* is the true guide in therapeutics.

*Fourth.*—The totality of the symptoms forms the only basis for the selection of the remedy, and the best results are attained by the use of the minimum dose of the single remedy in a potentiated form.

*Fifth.*—The alternating or combining of remedies in a prescription is non-homœopathic.

*Sixth.*—The suppression of symptoms by crude drugs in large doses and by local treatment is non-homœopathic.

*Seventh.*—Mechanical appliances are admissible only when mechanical conditions are to be overcome.

*Eighth.*—We deprecate any practice which tends to the suppression of symptoms, inasmuch as it injures the patient and renders difficult the selection of the specific remedy.

*Ninth.*—We disavow all connection with that practice which, under the guise of Homœopathy, is at variance with the law of similars and its conditions, as deduced by Samuel Hahnemann.

*Tenth.*—In order to publicly declare our allegiance to the homœopathic law, we have associated ourselves, and have organized under the name of the International Hahnemannian Association.

Guided by the foregoing declaration, we are enabled to judge of the merits of homœopathic therapeutics, and the demand therefor in any case, and of the occasion for bringing to our aid proper surgical assistance for the relief of the suffering, or a conjunction of the two when necessary.

Turning to the first, second, third, and fourth sections of the

*Organon*, we find a clear statement of the duty of the physician, also of the necessity for mechanical-surgical procedure in section 7.

The law of the land, and the curriculum of the medical schools require of the physician a knowledge of anatomy, physiology, pathology, materia medica, and therapeutics, also of obstetrics and of surgery, before he or she may enter upon the duties of the profession. It is true, some make a specialty of a particular department of medicine, obstetrics or surgery, but it many, *many* times happens, in small towns and in country practice, that the physician is called upon to treat fractures, traumatic conditions, hemorrhages, abscesses, tumors; perform paracentesis, catheterism, etc., and is expected to be *prepared* for such work. If he be a homœopathician, all the better, for certain constitutional symptoms often arise, acute inflammatory or neuralgic conditions precede or follow operative measures that are nicely and better controlled by homœopathic medication.

I think we who are radical in our views of what constitutes pure Homœopathy and sticklers for principle, err when we attempt to treat many cases in strict conformity to our declaration of principles. It often happens that homœopathic treatment will not cure the case until surgery steps in to remove the impediment, and this, too, in both acute and chronic diseases. I will cite a case by way of illustration, by no means an isolated case:

When practicing in Fulton, N. Y., back in the sixties, a lady of fifty years, fat, inclined to easy perspiration, sour eructations in the morning and after eating, also after taking milk, dry cough at night followed by loose, salty, yellow expectoration, morning asthma, and dyspnœa on slight exertion, cold, damp hands and feet, depression of spirits with apprehension of pending misfortune, came to me for advice. She also had scirrhus of the right breast, the breast being large, pendulous, quite hard and nodulated, and was obliged to carry it in a sling. She was subject to sharp lancinating pain in the breast, the pain often streaking down the arm and into neck. She lost much sleep and began to have night-sweats, also to lose flesh.

Under the action of Calc-carb.$^{200}$, and later on of the 5,000th potency, there was marked diminution of the constitutional symptoms but no real improvement of the local. The tumor increased in size, the nipple entirely retracted, and ulceration of the skin over the most prominent nodule of the tumor now took place. The ulceration increased, dipping into the body of the tumor and established a foul, sanious discharge with occasional hemorrhage. At this stage, six weeks from first prescription, I advised immediate extirpation of the entire breast. So on one of the coldest, stormy days of February or March, in 1865, assisted by Drs. MacManus and Fowler, of Oswego, and Dr. Chaffee, of Fairport, N. Y., removed the entire breast. The lady made a nice recovery; two-thirds of the superior line of wound healed by the first intention. The entire tumor weighed not far from eight pounds. In some three or four weeks she was able to resume work. The recovery seemed to be complete, and so far as I know she never had a return of the malady, for I kept posted as to her condition more than a decade.

The lesson to be learned from this and similar cases is: *that Homœopathy and surgery are intimately and philosophically related—are handmaids;* that remedies selected under homœopathic law put patients in the best condition for operative measures and give immunity from subsequent ill; also that structural changes and lesions frequently limit the curative action of remedies, the organism resuming its integrity after the removal of the altered condition by operative measures.

As a rule, in the treatment of cases made complex by an injury, or by certain organic changes, operative measures are not resorted to early enough, hence Homœopathy is likely to be the loser. To avoid this, and to maintain the philosophical relation of homœopathic therapeutics to surgery, there must be co-ordinate harmonious action, each coming to the assistance of the other, at the right time and by the right means.

It can be conclusively shown that only homœopathic therapeutics are related to, and in harmony with pathological symptomatological conditions—and philosophically so. Allopathic, antipathic measures are but the opposers, the brute force suppressors of natural law as exhibited in morbid conditions : hence, they are without chart, without warrant, unphilosophical, injurious !

There should be no conflict of opinion among us as to the policy or necessity of maintaining a Bureau of Surgery in our organization nor of encouraging surgeons of our school. There may be at times a question as to the necessity for operative measures, but such question can be easily set at rest by adopting the rule or statement made by our esteemed President, Dr. James B. Bell, on page 6 of his printed circular to "The Censors and Members of the International Hahnemannian Association," in which he says : "*We will never operate while there is a reasonable opportunity or expectation for the action of the specific remedy.*"

Hahnemann, in a foot-note to section 7 of his *Organon,* says: "It is taken for granted that every intelligent physician will commence by removing this 'causa-occasionalis ;' then the indisposition usually yields of itself. Thus it is necessary to remove flowers from the room when their odors occasion paroxysms of fainting, hysteria ; to extract from the eye the foreign substance that occasions ophthalmia ; remove the tight bandages from a wounded limb which threatens gangrene, and apply others more suitable ; lay bare and tie up a wounded artery where hemorrhage produces fainting ; evacuate the berries of Belladonna which may have been swallowed by vomiting ; extract the foreign particles which have introduced themselves into the openings of the body—the nose, pharynx, ears, urethra, rectum, vagina—grind down stone in the bladder ; open the imperforate anus of the new-born infant, etc."

Thus we see that Hahnemann, even at the opening of *The Organon,* recognized the occasional presence of and the necessity for surgical interference in removing a causa-occasionalis. It is also true that Hannemann in the foot-note to section 7 did not intend to limit the occasions for interference by mechanical methods to the examples then and there quoted. Had he lived until to-day, he probably would not condemn the use of water, hot or cold, nor of "Peroxide of Hydrogen," nor perhaps of some of the phenol group, when used as aseptics.

The fact is that certain measures, *chemical*, may occasionally be resorted to in the treatment of injuries, solutions of continuity, entirely legitimate, philosophical, and harmonious with our creed.

The philosophical relation of homœopathic therapeutics to surgery—and *vice versa*—must be determined by the symptoms and necessity of the case and on the basis of reason and experience. There are many cases illustrative of the philosophical relation of homœopathic therapeutics to surgery—a few I will name:

*First.*—The inflammatory and neuralgic conditions following fracture or dislocation of bone.

*Second.*—The inflammation of soft tissues after injuries, including erysipelas, erythema, etc.

*Third.*—Abscesses and other suppurative conditions.

*Fourth.*—Ulcerations.

*Fifth.*—All symptomatic fevers.

*Sixth.*—Hemorrhages of several kinds, particularly such as are associated with malignant disease, ulcerations, hemorrhoids, the bleeding from small wounds, polypi.

*Seventh.*—Cachectic and hectic conditions, after operation.

*Eighth.*—Gangrene; sloughing of tissue.

*Ninth.*—Ostitis, periostitis.

*Tenth.*—Collapse after injury.

*Eleventh*—Obstetrical complications.

*Twelfth*—The removal of tumors; opening and irrigation of abscesses; drainage, etc.

Other citations might be made—surgical and therapeutical—illustrating the harmonious relation of the two, but these must suffice. To sum up the whole matter, it may be said: That the interference of operative measures must be left to the discretion of the practitioner, based upon knowledge and experience, guided by reason and necessity. When this is done the relation of therapeutics and surgery will be *philosophical* and harmonious.

At this point it was my intention to close this paper, but on looking over the article, and in the light of the controversy that seems inevitable and likely to grow out of the position of our very worthy and highly esteemed President, Dr. James B. Bell, in his published letter to the members of this Association, and of some letters I have lately received on the subject, it seems necessary to add a few sentences relative to resolutions second and third of the Declaration of Principles.

If resolution second can be construed to apply to surgical procedure, it refers to caustic, astringent, styptic, aseptic, and antiseptic applications mainly; also to anæsthesia, poulticing, etc. Now, cases *may* arise where, in order to destroy a malignant growth, or to promote adhesive inflammation caustic may be used.

Tannin, Hamamelis-virg., Liquor Ferri-persulph. may be used to check hemorrhage from closed wounds, and the practice be rational and successful. No one claims such treatment to be homœopathic, but the same may be said of much if not all surgery. The free use of cool or hot water, poultices, the use of peroxide of hydrogen, of soap, of hydronaphthal in irrigating and cleansing wounds are non-homœopathic, but often necessary for the sake of cleanliness, and the spirit of Homœopathy is not violated by such use; *rather* is it maintained by it.

Resolution third of the Declaration of Principles is to be interpreted in the light of the full meaning of the term "mechanical," when defined by the popular understanding and by experts. Webster defines the word "mechanical" as follows:

"Pertaining to, governed by, or in accordance with mechanics, or the laws of motion; depending upon mechanism or machinery."

"That part of *mechanics* which considers the action of forces in producing rest or equilibrium is called *statics;* that which relates to such action in producing motion is called *dynamics.*"

"The term *mechanics* includes the action of forces on all bodies, whether solid, liquid, or gaseous."

If we accept the above definitions as sound, it is easy to see that very few surgeons inside this Association violate the spirit, or even the letter of our principles in their practice. Large liberty, wide latitude are given them in a surgical point of view. Surgical operations are dynamic or statical, and whether one or both may be not only consistent with mechanics, but with homœopathics.

I am but a humble member of this Association, yet will not be behind any in sustaining our principles upon logical, philosophic bases. But allow me to express sorrow that so little interest seems to be taken in things that relate to the Bureau of Surgery, and that the surgeons of our school and of this Association should be so severely catechised for their conscientious and legitimate work in curing the sick—or at least in *trying* to do so. Lady and gentleman practitioners, if the work of ostracism or of expulsion must begin, have you reckoned the consequences, and do you know where it will stop? To be pure is *right*, to weed out tares is well, but "in weeding out the tares *be mindful, lest you tear out wheat also!*"

---

Dr. B. Fincke was appointed Chairman of the Bureau of Homœopathic Philosophy for the ensuing year.

The following corrections are desired to be made in regard to the Transactions of 1891.

The remarks of the President on page 338 at the close of the paper, "Alternate Action," should be placed on page 342 at the close of the paper, "Inversion of Symptoms, Repetition."

The draft of a Declaration of Principles, presented by Dr. Fincke and referred to a committee, was inadvertently omitted from the Transactions of 1891. It is here given in full.

